# The Value of Ultrasound Combined With Thromboelastography in Dynamic Monitoring of Thrombosis in Patients on Maintenance Hemodialysis With Arteriovenous Fistulas

**DOI:** 10.7759/cureus.97864

**Published:** 2025-11-26

**Authors:** Liuyi Chi, Fubin Zhang, Jie Shen, Penghui Wang, Runming Zhong, Weili Lin, Hui Xia, Jinchun Chen

**Affiliations:** 1 Ultrasound Department, Rui'an People's Hospital, Rui'an, CHN; 2 Radiology Department, Rui'an People's Hospital, Rui'an, CHN

**Keywords:** arteriovenous fistula, dynamic monitoring, maintenance hemodialysis, thromboelastography, ultrasound

## Abstract

Objective: The objective of this study was to explore the value of dynamic detection of thrombelastogram (TEG) combined with ultrasound in thrombus formation in patients on maintenance hemodialysis (MHD) with arteriovenous fistula (AVF).

Methods: A total of 98 patients undergoing MHD with an upper limb forearm AVF who met the diagnostic criteria were randomly selected from our hospital. Three cases were lost to follow-up, resulting in 95 participants. According to the blood flow at the fistula site, there were 26 cases in the abnormal blood flow group and 69 cases in the normal blood flow group. TEG and ultrasound examinations were performed on the same day before and after the fistula creation, once every two days, for a total of five consecutive tests; 3 mL of peripheral venous blood was drawn for TEG examination. The differences in TEG indicators (R value, K value, α angle, and MA value) between the two groups were compared. Linear regression and binary logistic regression were used to compare TEG indicators, ultrasound, and the combined diagnosis of abnormal blood flow at the fistula site, along with sensitivity and specificity, and the receiver operating characteristic (ROC) curve.

Results: The TEG indicators for the abnormal blood flow group and the normal blood flow group were 3.72±1.36 and 7.34±1.40, 0.72±0.34 and 2.08±0.44, 78.77±9.14 and 61.78±5.41, 74.96±10.63 and 58.63±5.68, respectively, with significant differences between the two groups (all P < 0.05). The variance inflation factor (VIF) for TEG indicators and ultrasound diagnostic single indicators was 3.627, 4.479, 3.400, 3.289, 3.9789 (all < 5), indicating no multicollinearity, while the combined diagnostic model had a VIF of 6.334 (> 5), indicating the presence of multicollinearity.

Binary logistic regression analysis showed that TEG indicators, ultrasound diagnosis, and combined diagnosis abnormalities (OR = 1.244, 1.202, 1.316, 1.374, 1.460, 1.547, all P < 0.001) were independent risk factors for abnormal blood flow at the AVF site. The sensitivity and specificity of TEG, ultrasound diagnosis, and combined diagnosis were 76.9% and 4.3%, 88.5% and 4.3%, 73.1% and 4.3%, 76.9% and 2.9%, 88.5% and 4.3%, 96.2% and 0.0%, respectively. The combined diagnosis had high sensitivity and low specificity, but the differences were not statistically significant (all P > 0.05). The area under the ROC curve for the combined diagnosis was significantly larger than that for TEG indicators and ultrasound diagnosis. The dialysis blood flow (ml/min) before and after treatment in the abnormal blood flow group was 153.5±16.1 and 322.6±38.6, respectively, with significant differences between them (t=18.402, P < 0.05).

Conclusion: All TEG indicators in the abnormal blood flow group of MHD-AVF were higher than those in the normal blood flow group. TEG indicators and ultrasound examination in MHD-AVF patients are risk factors for abnormal blood flow at the fistula site. TEG combined with ultrasound has better collinearity, high sensitivity, low specificity, and the AUC is higher than that of separate TEG and ultrasound diagnoses. The combined diagnosis of TEG and ultrasound can improve the diagnostic rate of abnormal blood flow at the MHD-AVF fistula site.

## Introduction

Arteriovenous fistula (AVF) is the primary vascular access for patients undergoing maintenance hemodialysis (MHD); however, thrombosis is one of the main causes of its dysfunction, severely affecting dialysis effectiveness and patient quality of life. Currently, clinical assessment of AVF patency mainly depends on ultrasound, which has limitations in predicting early thrombosis. Thromboelastography (TEG), as a technology for dynamically monitoring blood coagulation, can comprehensively assess coagulation status, reflecting platelet function, thrombin generation, and fibrin formation [1].

While TEG has shown distinct advantages in diagnosing thrombotic conditions clinically, research remains limited both in China and internationally on the combined use of ultrasound and TEG for dynamic monitoring of AVF thrombosis in patients on MHD, and its clinical application value requires further exploration. Thus, this study aimed to dynamically monitor TEG and ultrasound parameters in patients on MHD after AVF surgery to investigate the predictive value of their combined diagnosis for thrombosis, providing grounds for early clinical intervention, optimizing AVF monitoring strategies, and may offer new insights for improving the prognosis of patients undergoing MHD.

## Materials and methods

This was a prospective study conducted at the Rui'an People's Hospital, Rui'an, Zhejiang, China, from October 30, 2022, to October 30, 2023. The study was approved by the Ethics Committee of Ruian People's Hospital (approval number: YJ2022050, dated September 26, 2022). All participants gave written informed consent.

Study population

Exclusion criteria include severe liver disease, use of anticoagulants, tumors, coagulation disorders, and other systemic diseases. A total of 98 patients with chronic kidney disease undergoing MHD with upper arm forearm AVF who met the diagnostic criteria were randomly selected from our hospital. Three cases lost to follow-up, and thus, a total of 95 cases were included in the study.

Based on whether blood flow slowed down or got blocked during hemodialysis, participants were divided into two groups: (i) Abnormal blood flow group (Group A), which had 26 cases, and (ii) Normal blood flow group (Group B), which had 69 cases.

Intervention

AVF Creation

AVF was performed by a physician at the level of deputy chief or above. Preoperative assessment for AVF creation included arterial inner diameter ≥ 1.5 mm, venous inner diameter ≥ 2 mm, and distance from the epidermis > 6 mm. Radial artery-cephalic vein fistula was selected, followed by disinfection and sterile draping. There was routine use of sodium heparin (batch number: 20220108; Tianjin Biochemical Pharmaceutical Co., Ltd, Tianjin, China) 100 U/Kg diluted with 5 ml of 0.9% saline, IV, once daily before arterial occlusion during surgery. Mature AVF assessment included brachial artery blood flow > 500 ml/minute in the area 5 cm above the elbow (as calculated by the following formula: Blood flow (ml/min) = area (cm^2^) × time average flow rate (cm/s) × 60), puncture segment venous inner diameter ≥ 5mm, and depth from the skin < 6mm.

Ultrasound Examination

The ultrasound machine used was a Phillips iE33 color Doppler ultrasound with a 5-15 MHz probe (Koninklijke Philips N.V., Amsterdam, Netherlands). The ultrasound examination was conducted by specialized personnel (attending physician or senior) from our research group. The examination started after the patient had undergone the stoma surgery, and was done every two days for 10 days (five sessions in total). Patients were supine with the arm abducted. The probe was held to avoid vessel compression. Parameters assessed included arterial inflow, fistula site, venous outflow patency, stenosis, occlusion, vessel wall thickness, and perivascular echogenicity. Positive findings were marked as 1, negative as 0.

Ultrasound criteria for mature AVF blood flow: Within 10 cm of the anastomosis, vein diameter ≥4 mm; within 7 cm of the anastomosis, vein diameter ≥5 mm; blood flow volume >500 mL/minute; skin depth <6 mm; lumen fully compressible under probe pressure; outflow vein exhibits an arterial-like low-resistance Doppler spectrum.

Ultrasound diagnostic criteria for AVF stenosis [2,3]: (i) Arterial inflow stenosis: Peak velocity at the fistula site/peak velocity in the inflow artery segment 2 cm upstream ≥3, (ii) Venous outflow stenosis: Peak velocity at the stenosis site/peak velocity in the venous segment 2 cm downstream ≥2, indicating stenosis ≥50%, and (iii) Anastomotic stenosis: Anastomosis diameter <2.5 mm + abnormal blood flow (normal diameter 3-5 mm);

Ultrasound diagnostic criteria for AVF thrombosis [4,5]: Solid hypoechoic or hyperechoic material within the lumen; venous lumen compressibility under probe pressure ruled out thrombosis, otherwise it was taken as an indication for fistula occlusion due to thrombosis.

TEG Testing

On the day before the stoma creation, 3 mL of peripheral vein blood was drawn, and it continued once every two days, for a total of five consecutive times. TEG® 5000 Hemostasis Analyzer System (Haemonetics Corp., Boston, Massachusetts, United States) and matching reagent kit were used. Parameters measured included reaction time (R) (normal range, 5-10 minutes), kinetics time (K) (normal range, 1-3 minutes), alpha angle (α angle) (normal range, 53°-72°), and maximum amplitude (MA) (normal range, 50-70 mm). Normal R, K, α angle, and MA values were marked as 0; abnormal values were marked as 1.

Diagnostic criteria for fistula stenosis/occlusion

We developed the following criteria for combined diagnosis: (i) Ultrasound detection of stenosis or occlusion, (ii) All four TEG parameters (R, K, α angle, MA) abnormal. Presence of one or both criteria confirmed diagnosis, marked as 1; otherwise marked as 0. For AVF occlusion diagnosis, we considered an extracorporeal blood flow rate <200 mL/minute.

Anticoagulation therapy

For anastomotic thrombosis, urokinase injection (batch no. 2021120623; Jilin Aodong Taonan Pharmaceutical Co., Ltd., Dunhua, Jilin, China), 50,000-500,000 IU (average 216,000 ± 131,000 IU), diluted with 5 mL of 0.9% saline, was injected into the vein 2-3 cm proximal to the thrombus. A tourniquet was applied 10 cm proximal to the thrombus before injection, followed by gentle massage of the thrombosed area. Ultrasound re-examination was performed after 10 minutes to assess thrombus dissolution. Successful thrombolysis was defined as dialysis blood flow >200 mL/minute. If thrombolysis failed, surgical revision was required.

Observations

Observations included: (i) Comparison of TEG parameters between the abnormal and normal blood flow groups, (ii) Correlation analysis between TEG and ultrasound parameters and abnormal dialysis blood flow, and (iii) ROC curves for TEG, ultrasound, and combined diagnostic parameters.

Statistical analysis

Statistical analysis was performed using SPSS 19.0 software. Continuous data were expressed as mean ± standard deviation. Group comparisons were done using paired t-test and one-way ANOVA. Categorical data were analyzed using chi-square or Fisher's exact test for small sample sizes (n < 5). The correlation between the two groups was assessed using linear regression and binary logistic regression. Statistical significance was set at *P* < 0.05.

## Results

A total of 95 participants were included in the study. Of this, 26 were in the Abnormal blood flow group (Group A) and 69 were in the Normal blood flow group (Group B). Group A had 12 male and 14 female participants aged between 40 and 79 years, with an average age of 60.1±9.1 years; the fistula duration was between 4 and 71 months, with an average of 35.7±12.6 months. Group B had 33 male and 36 female participants aged between 41 and 78 years, with an average age of 64.4±8.4 years; the fistula duration was between 5 and 73 months, with an average of 36.9±12.8 months. There were no significant differences in gender, age, or fistula duration between the two groups (all P>0.05). 

Thromboelastography analysis of the two groups

The R, K, α angle, and MA values in Group A and Group B were 3.72±1.36 and 7.34±1.40, 0.72±0.34 and 2.08±0.44, 78.77±9.14 and 61.78±5.41, and 74.96±10.63 and 58.63±5.68, respectively. All differences between groups were statistically significant (all *P* < 0.05), as shown in Table 1.

**Table 1:** Thromboelastography analysis of the two groups Two sets of means using paired data t-test R: reaction time; K: kinetics time; MA: maximum amplitude

Type	Count	R (minutes), mean±SD	K (minutes), mean±SD	ɑ angle (°), mean±SD	MA (mm), mean±SD
Abnormal blood flow group	26	3.72±1.36	0.72±0.34	78.77±9.14	74.96±10.63
Normal blood flow group	69	7.34±1.40	2.08±0.44	61.78±5.41	58.63±5.68
t value		9.473	14.627	8.609	7.177
P value		0.000	0.000	0.000	0.000

TEG, ultrasound, and combined diagnosis for detecting blood flow abnormalities in AVFs

R value, K value, α angle, MA value, and the variance inflation factor (VIF) of the ultrasonic diagnosis single indicators were 3.627, 4.479, 3.400, 3.289, and 3.979, respectively (all < 5), indicating no multicollinearity; the joint diagnostic model VIF was 6.334 (> 5), indicating the presence of multicollinearity.

The standard for maintaining blood flow at the AVF site is based on the blood flow (abnormal = 1, normal = 0). Binary logistic regression analysis shows that abnormal R value (OR = 1.244, P < 0.001), abnormal K value (OR = 1.202, P < 0.001), abnormal α angle (OR = 1.316, P < 0.001), abnormal MA value (OR = 1.374, P < 0.001), abnormal ultrasound diagnosis (OR = 1.460, P < 0.001), and abnormal combined diagnosis (OR = 1.547, P < 0.001) are all independent risk factors for abnormal blood flow at the AVF site (Table 2).

**Table 2:** Evaluation of TEG, ultrasound, and combined diagnosis for detecting blood flow abnormalities in AVFs of patients undergoing MHD Using linear regression and binary logistic regression analysis AVF: arteriovenous fistula; MHD: maintenance hemodialysis; TEG: thromboelastography; R: reaction time; K: kinetics time; MA: maximum amplitude

Type	β value	SE	Wald value	OR value	X^2^	P value	Collinearity diagnosis	
							tolerance	VIF
R value	-0.027	0.001	0.020	1.244	57.252	0.000	0.276	3.627
K value	-0.069	0.004	0.008	1.202	47.853	0.000	0.223	4.479
ɑ angle	0.002	0.000	0.001	1.316	48.564	0.000	0.294	3.400
MA value	0.003	0.000	0.000	1.374	52.393	0.000	0.304	3.289
Ultrasonic diagnosis	0.146	0.007	0.001	1.460	62.498	0.000	0.251	3.978
Joint Diagnosis	0.669	0.008	0.000	1.547	86.567	0.000	0.158	6.334

Sensitivity and specificity of TEG, ultrasound, and combined diagnosis for detecting blood flow abnormalities at AVF sites

The sensitivity versus specificity values were as follows: R value (76.9% vs 4.3%), K value (88.5% vs 4.3%), α-angle (73.1% vs 4.3%), MA (76.9% vs 2.9%), ultrasound diagnosis (88.5% vs 4.3%), and combined diagnosis (96.2% vs 0.0%). The combined diagnosis showed high sensitivity but low specificity. However, no statistically significant differences were observed in sensitivity/specificity when compared with R value, K value, α-angle, MA, or ultrasound diagnosis (all P > 0.05). Results are summarized in Table 3.

**Table 3:** Analysis of sensitivity and specificity of TEG parameters, ultrasound, and combined diagnosis in detecting blood flow abnormalities in AVF of patients undergoing MHD Count data were analyzed with the chi-square test, or Fisher's exact test when n < 5. AVF: arteriovenous fistula; MHD: maintenance hemodialysis; TEG: thromboelastography; R: reaction time; K: kinetics time; MA: maximum amplitude

Type	Count	False negative (example)	True positive (example)	False positive (example)	True negative (example)	Sensitivity (%)	Specificity (%)
R value	95	6	20	3	66	76.9	4.3
K value	95	3	23	3	66	88.5	4.3
ɑ angle	95	7	19	3	66	73.1	4.3
MA value	95	6	20	2	67	76.9	2.9
ultrasonic diagnosis	95	3	23	3	66	88.5	4.3
Joint Diagnosis	95	1	25	0	69	96.2	0.0

ROC analysis of TEG parameters, ultrasound, and combined diagnosis for detecting hemodialysis access dysfunction at the AVF site

**Figure 1:**
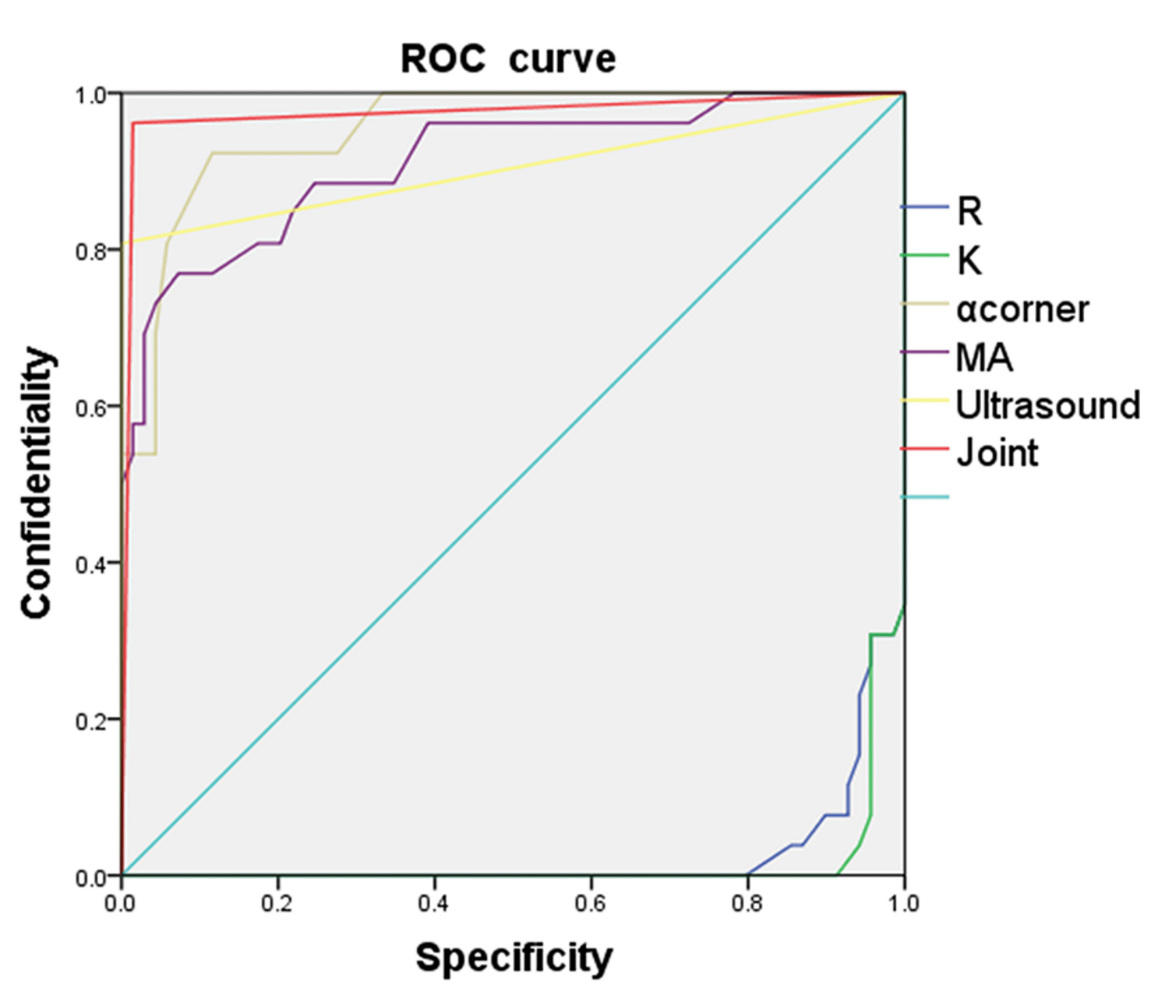
ROC curve analysis of TEG parameters, ultrasound, and combined diagnosis for detecting hemodialysis access dysfunction at the AVF site in patients undergoing MHD AVF: arteriovenous fistula; MHD: maintenance hemodialysis; TEG: thromboelastography; K: kinetics time; R: reaction time; MA: maximum amplitude; ROC: receiver operating characteristic

This study successfully established a Logistic regression model based on R value, K value, α angle, MA, ultrasound, and combined diagnosis. The area under the ROC curve (AUC) for R value, K value, α angle, MA, ultrasound, and combined diagnosis were 0.025, 0.015, 0.954, 0.912, 0.904, and 0.974, respectively. The AUC for combined diagnosis was significantly greater than that for R value, K value, α angle, MA, and ultrasound diagnosis (Figure 1).

Minute dialysis blood flow rate before and after treatment in Group A

The dialysis blood flow rates before and after treatment (ml/min) in Group A were 158.6±12.6 and 267.6±21.1, respectively. A paired t-test showed the difference was statistically significant (t=18.402, P<0.05), as shown in Table 4.

**Table 4:** Comparison of minute dialysis blood flow rate before and after treatment in Group A (N=26) Comparison of means between two groups using one-way t-test.

Type	Dialysis blood flow/minute (ml), mean±SD	t	P
Before treatment	153.5±16.1	18.402	0.000
After treatment	322.6±38.6		

## Discussion

Comparison of TEG between abnormal and normal blood flow groups at the AVF site in patients on MHD

The R value represents coagulation initiation time, while the K value reflects fibrin formation speed. Shortened R and K values indicate heightened coagulation system activation. Elevated fibrinogen (FIB) and factor VIII levels accelerate thrombin generation, consistent with TEG parameter changes [6]. Vascular endothelial injury increases tissue factor (TF) release, further activating the extrinsic coagulation pathway and promoting thrombus formation [7]. In this study, chronic kidney disease patients on MHD with abnormal AVF blood flow showed significantly lower R and K values than the normal group, likely stemming from enhanced coagulation factor activity and potentially elevated fibrinogen levels.

The α angle reflects fibrin formation rate, with increased values indicating accelerated fibrinogen-to-fibrin conversion. Uremic toxins (such as p-cresyl sulfate and indoxyl sulfate) in uremic patients promote fibrinogen cross-linking and enhance thrombus stability [8]. AVF stenosis patients exhibit elevated plasminogen activator inhibitor-1 (PAI-1) levels, inhibiting the fibrinolytic system and promoting fibrin deposition [9]. The abnormal blood flow group demonstrated significantly higher α angles, reflecting heightened fibrin formation and suggesting intensified fibrin polymerization.

The MA value represents maximum thrombus strength, primarily reflecting platelet count and function. The abnormal flow group showed significantly higher MA values, indicating enhanced platelet aggregation and activation. Upon stimulation, platelets rapidly express surface molecules that mediate adhesion between platelets, vascular endothelium, and inflammatory cells, promoting fibrin deposition and thrombus formation [10]. Platelet-driven thrombus formation involves adhesion, activation, and aggregation [11]. The elevated MA value underscores hyperactive platelet function in AVF blood flow abnormalities.

While TEG parameter changes offer new perspectives for early AVF dysfunction prediction, their clinical application remains controversial. TEG parameters may be influenced by factors like anemia, inflammation, or antiplatelet drugs, requiring comprehensive clinical assessment [12]. Some AVF stenosis patients show no significant TEG changes [13], suggesting alternative non-thrombotic mechanisms like vascular intimal hyperplasia. Future studies should expand sample sizes and incorporate multimodal analysis, including Doppler ultrasound and molecular markers (D-dimer, PAI-1). This study lends theoretical support for early AVF dysfunction identification and personalized anticoagulation therapy, but clinical utility requires further validation.

Value of TEG, ultrasound, and combined diagnosis in assessing AVF flow abnormalities in patients on MHD

This study shows that the VIF for R value, K value, α angle, MA value, and ultrasound diagnostic single indicators are 3.627, 4.479, 3.400, 3.289, and 3.9789 (all <5), indicating no multicollinearity, while the combined diagnostic model has a VIF of 6.334 (>5), indicating the presence of multicollinearity. TEG can comprehensively assess coagulation function, including coagulation initiation (R value), fibrin formation (K value, α angle), and platelet function (MA value). TEG is sensitive to hypercoagulable states associated with thromboembolic events [14]. Elevated α angle and MA value are associated with excessive platelet activation, which can promote microthrombus formation [15]. Each parameter of TEG reflects different stages of coagulation [16]. The degree of stenosis measured by ultrasound is often highly correlated with clinical thrombotic markers such as D-dimer [17], and sometimes ultrasound suggests stenosis at the anastomotic stoma, indicating surgical technique errors or intimal hyperplasia [18]. The role of ultrasound in early identification of AVF dysfunction [19]. The combined diagnosis integrates multiple information, bringing more stability to the statistical model.

Binary logistic regression analysis shows that R value, K value, α angle, MA value, ultrasound diagnosis, and combined diagnosis abnormalities (OR = 1.244, 1.202, 1.316, 1.374, 1.460, 1.547, all P < 0.001) are independent risk factors for abnormal blood flow at the AVF stoma. Color ultrasound can accurately display vascular blood flow status, lumen structure, and thrombus formation [20]. Single-modal methods may reduce sensitivity and specificity for clinical conditions with different pathophysiology [21]. TEG is sensitive to hypercoagulable states associated with thromboembolic events [22]. TEG is an effective method for identifying hypercoagulable states or reflecting coagulation changes [23]. Relying solely on ultrasound examination or TEG parameters, some patients with normal ultrasound and TEG still exhibit abnormal blood flow at the stoma. Diagnosing abnormal blood flow at the hemodialysis AVF stoma requires optimizing multimodal models and combining vascular biological markers (such as endothelial injury factors) to achieve higher predictive accuracy.

Sensitivity, specificity, and ROC curve analysis of TEG, ultrasound, and combined diagnosis in detecting blood flow abnormalities at the AVF site in patients on MHD

The results demonstrated that the combined diagnostic method had high sensitivity but low specificity in detecting hemodialysis fistula blood flow abnormalities; however, it showed no statistically significant difference when compared to R value, K value, α angle, maximum amplitude (MA), or ultrasound diagnosis (with all comparisons showing P > 0.05). Further studies with larger sample sizes are warranted.

This study used ROC curve analysis to confirm that the combined diagnostic strategy shows clear advantages for predicting blood flow abnormalities at the AVF site in patients undergoing MHD. The AUC for the combined approach was much higher than that for single-parameter diagnosis (R value, K value, α angle, MA) or ultrasound diagnosis alone.

The combined strategy works better because it looks at multiple underlying disease processes. AVF dysfunction is caused by multiple factors working together, including coagulation abnormalities, vascular remodeling, and hemodynamic changes. Our results show that the combined approach provides a full picture of AVF function by integrating coagulation function (R value, K value, α angle, MA) and vascular ultrasound assessment. It improves early detection of blood flow abnormalities in AVF dysfunction. At the same time, this method has its downside, including higher costs and more complex procedures.

Comparison of dialysis blood flow rate before and after treatment in the abnormal blood flow group

The results of this study show that early urokinase treatment for AVF blood flow abnormalities in patients on MHD yielded significant improvements. Before treatment, the patients' dialysis blood flow rate was only 158.6 ± 12.6 mL/minute, far below the clinically recommended optimal level (typically requiring an ultrafiltration flow rate ≥200 mL/minute) [24]. This insufficient flow rate hindered dialysis efficiency, leading to reduced urea clearance (Kt/V), accumulation of middle- and large-molecular toxins such as β2-microglobulin, and a significantly increased risk of complications related to inadequate dialysis, including anemia that is hard to treat, malnutrition, and cardiovascular events [25]. After treatment, the blood flow rate substantially improved to 267.6 ± 21.1 mL/minute (t = 18.402, P < 0.05), with both statistical and clinical significance.

The marked increase in blood flow primarily indicates a fundamental improvement in dialysis adequacy. Adequate blood flow is the foundation for maintaining effective solute clearance and a prerequisite for high-quality dialysis therapy. Correcting AVF stenosis restores the dialysis blood flow to optimal levels, significantly improving patients' short-term biochemical indicators and long-term survival outcomes [26].

The restoration of fistula function greatly improves patients' quality of life. Insufficient dialysis blood flow is often the main cause of repeated puncture difficulties, pressure alarms during dialysis, or even pump failures due to clotting, causing distress and anxiety. Effective intervention ensures smoother dialysis sessions by maintaining blood flow, reducing treatment interruptions, and enhancing patients' compliance and confidence in therapy [27].

From a pathophysiological perspective, abnormal fistula blood flow is frequently caused by proliferative stenosis at the anastomosis or venous outflow tract. The substantial post-treatment increase in dialysis blood flow confirms that interventions (e.g., percutaneous transluminal angioplasty (PTA)) successfully relieved localized organic stenosis, reduced vascular resistance, and restored normal hemodynamics. This not only improves dialysis efficiency but also helps delay the recurrence of fistula stenosis. Angioplasty effectively addresses intimal hyperplastic lesions, restores lumen diameter, and improves blood flow dynamics [28].

In summary, regular ultrasound and TEG monitoring for AVF fistula blood flow abnormalities in patients on MDH allows early detection and intervention for stenosis, effectively and significantly improving dialysis blood flow. This is crucial for ensuring dialysis adequacy, enhancing patient prognosis, and improving quality of life. This underscores the critical role of regular fistula function monitoring (e.g., Doppler ultrasound, TEG) and the establishment of a systematic "monitor-diagnose-treat" approach in hemodialysis management.

Significance and limitations

In MHD patients, stenosis at the AVF fistula site is typically diagnosed using abnormal hemodialysis flow as the gold standard, combined with ultrasound detection of thrombosis. However, this approach may result in irreversible outcomes for some patients if fistula obstruction treatment fails. The results indicate that combining TEG with ultrasound may improve diagnostic efficiency for abnormal hemodialysis flow in AV fistulas of MHD patients. This highlights the need for more accurate diagnostic methods. However, due to the limited sample size in this study, further validation with larger datasets is required.

## Conclusions

In patients on MDH, the R, K, α angle, and MA values in the group with abnormal blood flow were higher than those in the group with normal blood flow. Abnormal TEG parameters in MHD-AVF patients were associated with blood flow abnormalities at the fistula site. The combined diagnosis based on TEG and ultrasound showed better collinearity, high sensitivity, low specificity, and a higher AUC compared to independent diagnoses by TEG or ultrasound. The combined diagnosis may enhance the detection rate of blood flow abnormalities at the AVF site in patients on MHD.
